# Complete Chloroplast Genome of Four Thai Native *Dioscorea* Species: Structural, Comparative and Phylogenetic Analyses

**DOI:** 10.3390/genes14030703

**Published:** 2023-03-12

**Authors:** Warin Wonok, Runglawan Sudmoon, Tawatchai Tanee, Shiou Yih Lee, Arunrat Chaveerach

**Affiliations:** 1 Department of Biology, Faculty of Science, Khon Kaen University, Khon Kaen 40002, Thailand; 2 Faculty of Law, Khon Kaen University, Khon Kaen 40002, Thailand; 3 Faculty of Environment and Resource Studies, Mahasarakham University, Maha Sarakham 44150, Thailand; 4 Faculty of Health and Life Sciences, INTI International University, Nilai 71800, Negeri Sembilan, Malaysia

**Keywords:** chloroplast genome, *Dioscorea*, next-generation, phylogenetic analysis

## Abstract

The chloroplast genomes of *Dioscorea brevipetiolata*, *D*. *depauperata*, *D*. *glabra*, and *D*. *pyrifolia* are 153,370–153,503 bp in size. A total of 113 genes were predicted, including 79 protein-coding sequences (CDS), 30 tRNA, and four rRNA genes. The overall GC content for all four species was 37%. Only mono-, di-, and trinucleotides were present in the genome. Genes adjacent to the junction borders were similar in all species analyzed. Eight distinct indel variations were detected in the chloroplast genome alignment of 24 *Dioscorea* species. At a cut-off point of Pi = 0.03, a sliding window analysis based on 25 chloroplast genome sequences of *Dioscorea* species revealed three highly variable regions, which included three CDS (*trn*C, *ycf*1, and *rpl*32), as well as an intergenic spacer region, *ndh*F-*rpl*32. A phylogenetic tree based on the complete chloroplast genome sequence displayed an almost fully resolved relationship in *Dioscorea*. However, *D. brevipetiolata*, *D. depauperata*, and *D. glabra* were clustered together with *D. alata,* while *D. pyrifolia* was closely related to *D. aspersa*. As *Dioscorea* is a diverse genus, genome data generated in this study may contribute to a better understanding of the genetic identity of these species, which would be useful for future taxonomic work of *Dioscorea*.

## 1. Introduction

*Dioscorea* L. is the largest genus in Dioscoreaceae, containing approximately 600 recorded species, widely distributed in the Southeast Asia, Africa, Central America, South America, and other tropical and subtropical regions [1,2,3]. Members of *Dioscorea* are generally known as yams, an important vegetatively-reproducing tuber crop that is a good subsistence starch crop [4,5]. While many *Dioscorea* species are part of a staple diet in many countries, some of them are non-edible, as they contain toxic compounds [6]. Among them, many are identified as good natural resources with medicinal properties [7,8,9,10]. However, due to it being a diverse genus, identification and classification of *Dioscorea* species has been a challenge to taxonomists; the genus is dioecious, has small flowers, and comes with great morphological variations [11,12].

To shed light on the taxonomic status of the species in this complicated genus via molecular approaches, several phylogenetic studies have been carried out using DNA fingerprinting techniques, such as amplified fragment length polymorphism [13], polymerase chain reaction- restriction fragment length polymorphism [14], random amplified polymorphic DNA [15], and simple sequence repeat [16], as well as the use of short gene loci derived from nuclear DNA, Pgi [17] and Xdh [18], and chloroplast DNA (cpDNA), *atp*B-*rbc*L, *psa*A-*ycf*3, *rbc*L, *rpl*32-*trn*L, *mat*K, *trn*H-*psb*A, and *trn*L-*trn*F [11,12,19,20,21]. Although molecular markers provide some information on the taxonomy of *Dioscorea*, phylogenetic analyses are low resolution due to the limited data. Further studies to find high resolution molecular markers at the species level which lead to successful identification and phylogeny in the genus *Dioscorea,* are necessary [22]. Furthermore, the effort to perform molecular identification of *Dioscorea* species has been on-going [23,24,25]. Eventually, a study that utilized the highly variable regions in the cp genome of *Dioscorea* was proposed as a potentially useful marker for species delimitation and species identification among members of the complicated genus [3]. Despite the fact that studies on DNA barcoding in *Dioscorea* have been carried out to evaluate a suitable DNA barcode to discriminate the closely related species, the findings were inconclusive—only a limited number of samples were included in the study [26,27]. Note that *Dioscorea* is a diverse genus; thus, the work to barcode all species could be tedious and costly. For that reason, it is wise to look into informative sites in the cp genomes to aid in the barcoding effort of *Dioscorea*.

The rapid development of next-generation sequencing (NGS) platforms and bioinformatics tools in the last two decades has allowed the assembly and characterization of long sequences into complete organellar genomes to be conducted with ease [28,29]. In general, the chloroplast (cp) genome in angiosperms consists of a typical quadripartite structure, containing a pair of inverted repeats (IRs) that are separated by large single-copy (LSC) and small single-copy (SSC) regions [30]. The cp genome is generally maternally inherited, and has a genome size between 120 k and 170 k bp in length [22]. Its low rates of nucleotide substitutions and recombination make it suitable for use in phylogenetic studies of higher plants, thus resolving the complex evolutionary relationships in complicated genera [31,32]. On the other hand, complete cp genome sequences also enable researchers to understand various biological disciplines in plants, including gene families and functions, genome structure and evolution, phylogenomic relationship, etc. [33,34].

Using cpDNA is much preferred by researchers in phylogenetic studies, as demonstrated in *Dioscorea*; yet, studies have shown that the complete cp genome could increase phylogenetic resolution at low taxonomic levels when compared to the use of short gene sequences [22,35,36]. Owing to the need to reveal the phylogenetic relationships in *Dioscorea* at cp genome level, so far approximately 55 cp genome sequences, representing 35 *Dioscorea* species, have been made available in the NCBI GenBank database (as of January 2023). Despite the relevant amount of cp genomes that have been sequenced, the number of cp genomes reported for *Dioscorea* species was still less than 10% of the total species recorded in the genus. To expand the genetic information of *Dioscorea*, in this study, we sequenced anew and assembled the cp genome of four *Dioscorea* species that are native to Thailand. The assembled cp genome sequences of *D. brevipetiolata*, *D. depauperata*, *D. glabra*, and *D. pyrifolia* were characterized, and comparison analyses were conducted between the four species and other closely-related species. As a potential source of medicinal properties, we also identified several highly variable regions in the cp genome that could be developed into DNA markers. Phylogenomic analyses were also carried out to reveal the molecular placement of these species at cp genome level.

## 2. Materials and Methods

### 2.1. Plant Materials and DNA Extraction

Fresh, young leaf samples of four species of *Dioscorea*, including *D. brevipetiolata* (Prain and Burkill), *D*. *depauperata* (Prain and Burkill), *D*. *glabra* (Roxb.), and *D*. *pyrifolia* (Kunth), were collected from the Khon Kaen and Udonthani provinces, Thailand. The plants were identified following the Flora of Thailand, 2009, Dioscoreaceae, by the corresponding author. Specimen vouchers were kept at the Department of Biology, Faculty of Science, Khon Kaen University (KKU), collector numbers A. Chaveerach 1031, 1031.1, 1034, 1034.1, 1035, 1035.1, 1040, and 1040.1, respectively. The leaf samples were immediately kept in Ziplock bags containing silica gel beads, prior to being transported back to the laboratory for DNA extraction. Total genomic DNA was extracted using a DNeasy Plant Mini Kit (QIAGEN, Hilden, Germany), based on the manufacturer’s protocol. DNA purity and quantity were estimated using a Qubit™ 4 Fluorometer (Thermo Fisher Scientific, Waltham, MA, USA).

### 2.2. Next-Generation Sequencing, Genome Assembly, and Gene Annotation

A 350 bp paired-end library was prepared using a TruSeq DNA Sample Prep Kit (Illumina, San Diego, CA, USA) to obtain 150 bp pair-end reads. Next-generation sequencing was performed on the four collected species with an Illumina NovaSeq platform (Illumina, USA). The NGS QC Toolkit was used to trim off the adapter sequences [37] and the plastid genome was visualized using OrganellaGenomeDRAW v.1.3.1 [38]. The assembled cp genome was annotated, and the inverted repeat junctions were identified using GeSeq v.2.03 [39]. The circular cp genome was visualized using OrganellaGenomeDRAW v.1.3.1. The four *Dioscorea* species sequences of the annotated cp genome were deposited in the NCBI GenBank database under the accession numbers OL638495–OL638498.

### 2.3. Large Repeats and Simple Sequence Repeats (SSRs) Analysis

Large repeats, including the forward, palindromic, reverse, and complement repeats, were identified using REPuter [40], in which the minimum repeat size was set at 30 bp and the Hamming distance was set at 3. The SSRs present in the cp genome were identified using MISA-web [41]. The minimum number of repeat parameters were set at 10, 6, 5, 5, 5, and 5 for mono-, di-, tri-, tetra-, penta-, and hexanucleotide motifs, respectively.

### 2.4. Comparative Genome and Nucleotide Diversity Analysis

The junctions of the inverted repeats for 25 species of *Dioscorea*, including *D. abyssinica*, *D. baya*, *D. brevipetiolata*, *D. burkilliana*, *D. cayennensis*, *D. collettii*, *D. depauperata*, *D. dumetorum*, *D. elephantipes*, *D. esculenta*, *D. glabra*, *D. hirtiflora*, *D. japonica*, *D. nipponica*, *D. persimilis*, *D. polystachya*, *D. praehensilis*, *D. pyrifolia*, *D. quinquelobata*, *D. rotundata*, *D. sagittifolia*, *D. schimperiana*, *D. togoensis*, *D. villosa*, and *D. zingiberensis*, were visualized using the IRscope program [42] and the genes adjacent to them were identified. To ensure consistency in the annotation of gene content, the 25 downloaded cp genome sequences of *Dioscorea* were reannotated using GeSeq v2.03 [39] prior to junction analysis. Interspecific variation of the 25 species of *Dioscorea* at the cp genome level, including the four obtained from this study, was analyzed using mVISTA [43,44] with the Shuffle-LAGAN mode [45]. The cp genome of *D. bulbifera* was selected as the reference genome (Supplementary Materials, Table S1). Nucleotide diversity (Pi) in the LSC, SSC, and IR regions of the 25 species of *Dioscorea* was estimated using DnaSP v.6 [46]. The window length was set at 1000 bp, and 500 bp was selected for step size. The numbers of polymorphic sites and parsimony informative sites were also calculated.

### 2.5. Phylogenetic Reconstruction

Phylogenetic analysis was carried out based on the complete cp genome sequences of 37 species of Dioscoreaceae. Ten species—*Burmania coelestis*, *B*. *cryptopetala*, and *B*. *disticha* of Burmaniaceae, Diocoreales, as well as *Croomia heterosepala*, *C*. *japonica*, *C*. *pauciflora*, *Stamonia japonica*, *S*. *mairei*, *S*. *tuberosa*, and *S*. *sessilifolia* of Stemonaceae, Pandanales—were included as outgroups (Supplementary Materials, Table S1). All sequences were prepared by MEGA-X [47]. Multiple sequence alignment was performed using MAFFT v.7 [48] and phylogenetic trees were reconstructed based on two methods, maximum likelihood (ML) [49] and Bayesian inference (BI) [50]. The maximum likelihood was constructed using RAxML-HPC2 on XSEDE using a generalized-time-reversible (GTR) model with gamma (+G), and 1000 bootstrap replications were selected; for BI, the BI tree was constructed using MrBayes on XSEDE v.3.2.7a. A Markov chain Monte Carlo (MCMC) analysis was run for two million generations (Ngen = 2,000,000), with trees sampled every 100 generations. Both the ML and BI analyses were conducted using the pipelines available in the Cyberinfrastructure for Phylogenetic Research (CIPRES) Science Gateway v.3.3 [51]. Resulting trees were visualized using FigTree version 1.4.4 [52].

## 3. Results

### 3.1. Chloroplast Genome Structure of Dioscorea

**Figure 1:**
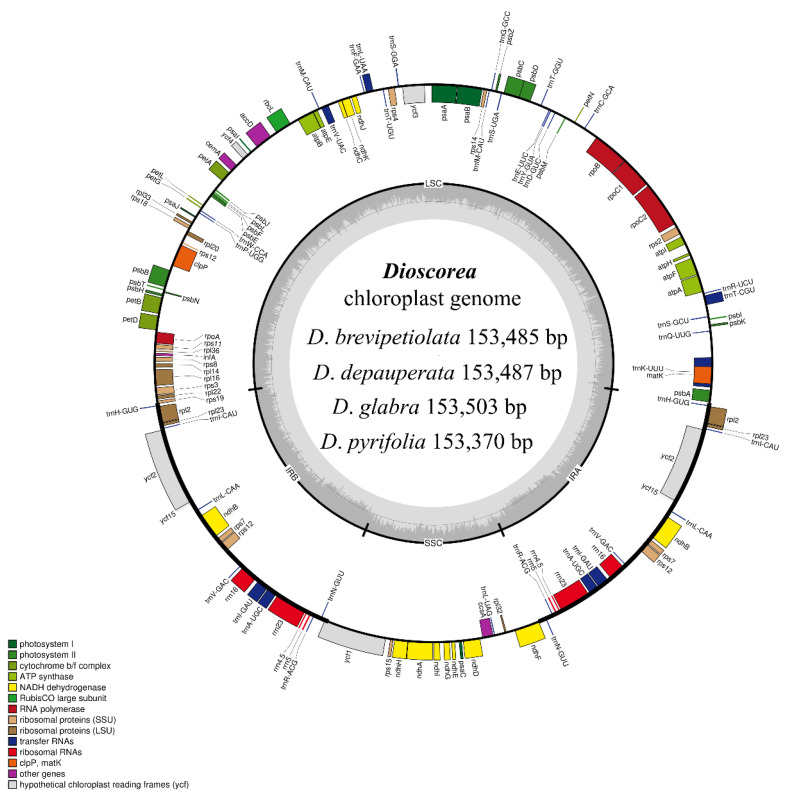
Genome structure and gene map of the four studied species, *Dioscorea brevipetiolata*, *D*. *depauperata*, *D*. *glabra*, and *D*. *pyrifolia*. The inside and outside circle genes are transcribed clockwise and counter-clockwise, respectively. The color codes represent different functional groups of the genes. The thick black lines indicate boundaries of the inverted repeats (IRA and IRB), divided between the LSC and SSC regions.

The complete cp genomes of the four species of *Dioscorea* showed a typical quadripartite structure in a circular form (Figure 1). The cp genomes were each comprised of a pair of inverted repeats (IRs), which were located between the large single-copy (LSC) and small single-copy (SSC) regions. The cp genome sizes varied from 153,370 bp (*D*. *pyrifolia*) to 153,503 bp (*D*. *glabra*). All four cp genomes were predicted to have the same total number of genes, which was 113, including 79 protein-coding (CDS), 30 tRNA, and four rRNA genes. The GC content of the four cp genomes obtained from this study was identical, and was 37% (Table 1). Groups of genes, functions of genes, and gene names are listed in Table 2. Among these genes, 18 of them were duplicated in the IR region, including *trn*H-GUG, *rpl*2, *rpl*23, *trn*I-CAU, *ycf*2, *ycf*15, *trn*L-CAA, *ndh*B, *rps*7, *trn*V-GAC, *rrn*16, *trn*I-GAU, *trn*A-UGC, *rrn*23, *rrn*4.5, *rrn*5, *trn*R-ACG, and *trn*N-GUU (Supplementary Materials, Table S2). A total of 19 genes contained introns, of which *trn*K-UUU had 2585 introns (*D*. *brevipetiolata*), 2586 introns (*D*. *depauperata*), 2604 introns (*D*. *glabra*), or 2577 introns (*D*. *pyrifolia*), *ycf*3 and *clp*P contained two introns, and *trn*T-CGU, *atp*F, *rpo*C1, *trn*L-UAA, *trn*V-UAC, *pet*B, *pet*D, *rpl*16, *rpl*2, *ndh*B, *rps*12, *trn*I-GAU, *trn*A-UGC, and *ndh*A each contained one intron (Supplementary Materials, Table S3).

### 3.2. Repeat Sequences and SSR Analysis

**Figure 2:**
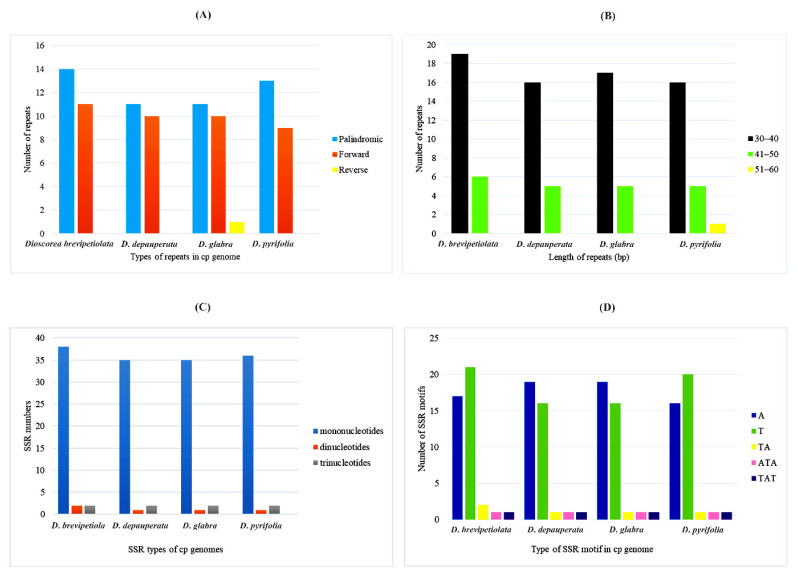
Large repeated sequences and simple sequence repeats in four *Dioscorea* cp genomes; the three repeat types, including palindromic, forward, and reverse (**A**); length group of repeat sequences (**B**); the three types of SSRs in *Dioscorea* cp genomes, including mononucleotides, dinucleotides, and trinucleotides (**C**); and the number of identified SSR motifs in different repeat types (**D**).

A total of 90 large repeats were detected in four cp genome sequences, of which 11–14 were palindromic repeats and 9–11 were forward repeats. One large reverse repeat was identified, which was derived from *D*. *glabra*. The repeat length that was most abundant was 30–40 bp in length, followed by the length 41–50 bp. The repeat length that was recorded the least was 51–60 bp, of which only one was found in *D*. *pyrifolia* (Figure 2; Supplementary Materials, Table S4).

The SSR analysis of the four studied *Dioscorea* species revealed three SSRs: mono-, di-, and trinucleotides. Mononucleotides was the most-observed type in all four studied *Dioscorea* species, with A and T present, while C and G were absent. A type was found the most in *D*. *depauperata* and *D*. *glabra* at 19 SSRs, followed by *D*. *brevipetiolata* at 17 repeats and *D*. *pyrifolia* at 16 repeats. T type was found the most in *D*. *brevipetiolata* at 21 SSRs, followed by *D*. *pyrifolia*, *D*. *depauperata*, and *D*. *glabra* at 20, 16, and 16 SSRs, respectively. For dinucleotides, there was only TA in *D*. *brevipetiolata* with two SSRs, with *D*. *depauperata*, *D*. *glabra*, and *D*. *pyrifolia* at one SSR each. Concerning trinucleotides, there were ATA and TAT with one SSR in all four studied *Dioscorea* species (Figure 2; Supplementary Materials, Table S5).

### 3.3. IR Expansion and Contraction

**Figure 3:**
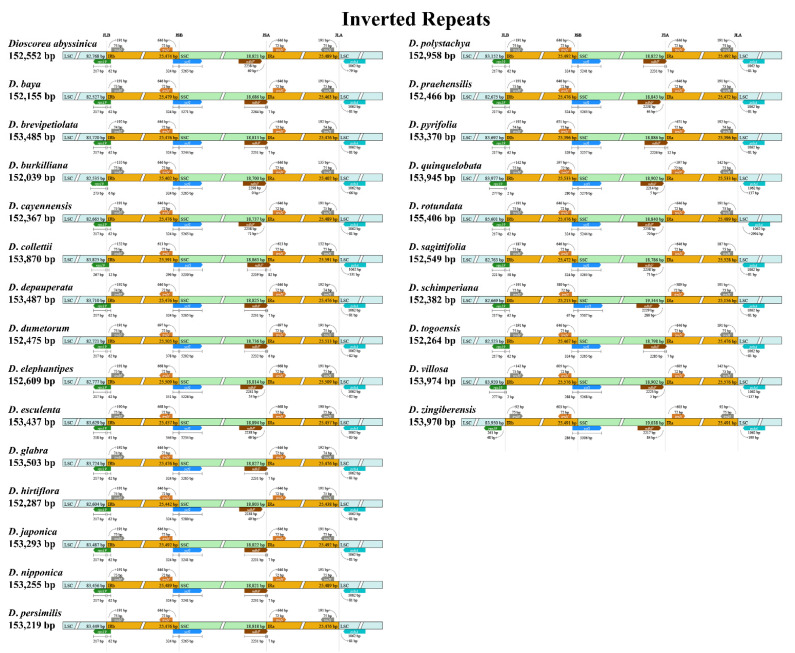
Comparisons of the border regions of LSC, SSC, and IR among 25 *Dioscorea* cp genomes; the boxes above and below the line indicate adjacent border genes. The figure only shows relative changes at or near the IR/SC borders, and is not to scale regarding sequence length.

There were four boundaries located between the LSC–IR and SSC–IR regions in all 25 cp genomes. In general, the genes adjacent to the boundaries were similar in all cp genomes analyzed (Figure 3). For the junction between the LSC and IRB regions (JLB), the *rps*19 gene was found crossing over from the IRB region into the LSC region for all species, except for *D. zingiberensis*; the *rps*19 gene of *D. zingiberensis* was placed in the LSC region and was 48 bp away from the boundary. On the other hand, the *trn*H genes, which were adjacent to JLB, were located in the IRB region in all species analyzed. For the junction between the SSC and IRB regions (JSB), two genes, *trn*N and *ycf*1, were placed next to the boundary. The t*rn*N gene was located in the IRB region, while *ycf*1 was identified crossing over from the IRB region into the SSC region for all species analyzed. For the junction between the SSC and IRA regions (JSA), *trn*N was found intact in the IRA region, while the *ndh*F gene that was located in the SSC region was found crossing over JSA in the cp genomes of 10 species of *Dioscorea*, including *D. baya*, *D. brevipetiolata*, *D. collettii*, *D. depauperata*, *D. dumentorum*, *D. glabra*, *D. japonica*, *D. nipponica*, *D. persimillis*, *D. polystachya*, *D. pyrifolia*, and *D. togoensis*. For the junction between the LSC and IRA regions (JLA), both the *trn*H and *psb*A genes were placed in the LSC and IRA regions, respectively.

### 3.4. Genomes Sequence Divergence among Dioscorea Species

**Figure 4:**
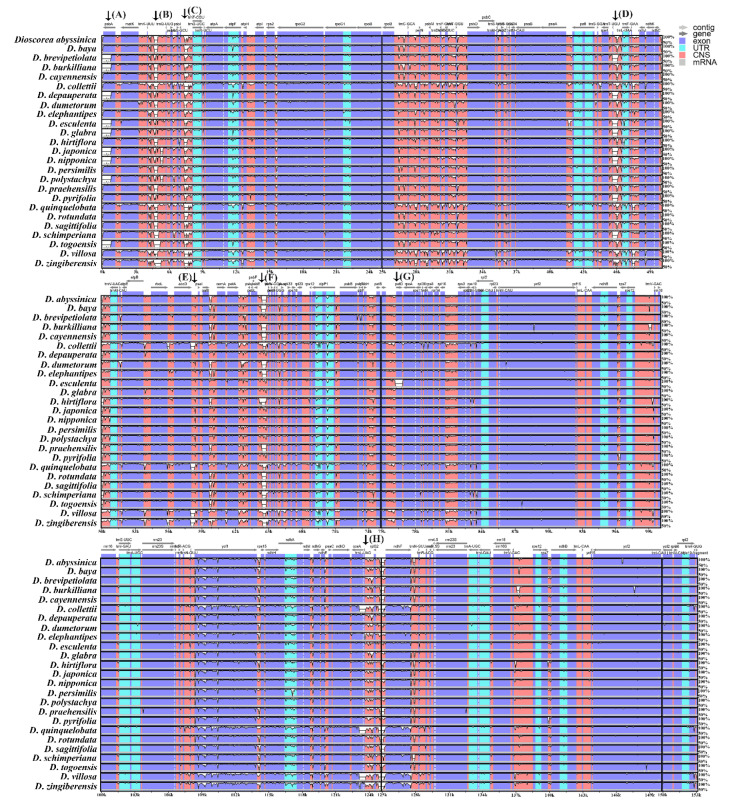
Comparative plots based on sequence identity of the 25 cp genomes of *Dioscorea* species, using *D*. *bulbifera* as the reference genome, constructed by mVISTA Software using Shuffle-LAGAN mode; the purple bars represent exons; pink bars represent conserved non-coding sequences (CNS); light-blue bars represent tRNA and rRNA regions; gray arrows above the aligned sequences indicate the genes and their orientations; the x-axis represents the number of bases in aligned sequences; the y-axis represents the percent identity within 50–100%; black arrows indicate regions which have a crucial divergence in variations located on LSC and SSC. Region with high variation include *psb*A (black arrow, A), *trn*K-UUU–*trn*Q-UUG (black arrow, B), *trn*S-GCU–*trn*G-UCC (black arrow, C), *trn*T-UGU–*trn*L-UAA (black arrow, D), *acc*D–*psa*I (black arrow, E), *psb*E–*pet*L (black arrow, F), *pet*D (black arrow, G), and *ccs*A–*trn*L-UAG–*rpl*32–*ndh*F (black arrow, H).

**Figure 5:**
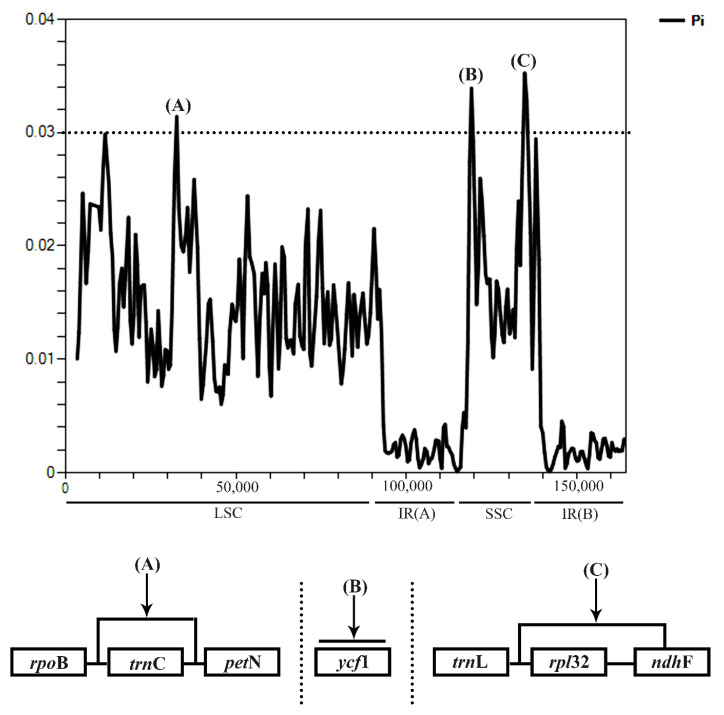
Nucleotide diversity (Pi) comparing the cp genome sequences of the 25 *Dioscorea* species using sliding window analysis (window length, 1000 bp; step size, 500 bp); the x-axis indicates the position of the midpoint; the y-axis indicates the nucleotide diversity of each window.

Genome comparison was analyzed in 25 *Dioscorea* cp genomes, including the four studied species and the 21 *Dioscorea* species derived from the NCBI database, with *D*. *bulbifera* for reference. The results indicated that the IR regions were more highly conserved than the LSC and SSC regions, with variations located on LSC and SSC. Eight variation gaps were observed in the cp genomes alignment; namely, *psb*A (black arrow, A), *trn*K-UUU through *trn*Q-UUG (black arrow, B), *trn*S-GCU through *trn*G-UCC (black arrow, C), *trn*T-UGU through *trn*L-UAA (black arrow, D), *acc*D through *psa*I (black arrow, E), *psb*E through *pet*L (black arrow, F), *pet*D (black arrow, G), and *ccs*A–*trn*L-UAG–*rpl*32–*ndh*F (black arrow, H). Variation gaps of *trn*K-UUU through *trn*Q-UUG, and *trn*S-GCU through *trn*G-UCC, were found in all *Dioscorea* cp genomes. Nine *Dioscorea* cp genomes had variation gaps at *psb*A and *trn*T-UGU through the *trn*L-UAA regions. Four *Dioscorea* cp genomes, *D*. *collettii*, *D*. *quinquelobata*, *D*. *villosa*, and *D*. *zingiberensis* had nucleotide divergence gaps at the *acc*D through *psa*I regions. Sixteen *Dioscorea* cp genomes had variation gaps at *psb*E through *pet*L region, while nucleotide divergence gaps in *pet*D were found only in *D*. *esculenta*. Three *Dioscorea* cp genomes, *D*. *colletii*, *D*. *quinquelobata*, and *D*. *villosa,* had distinct gaps in the *ccs*A–*trn*L-UAG–*rpl*32–*ndh*F region. These regions had more than 50% different nucleotide sequences from *D*. *bulbifera*, which was used for reference (Figure 4). Nucleotide diversity via sliding window analysis of the 25 cp genomes were compared in the LSC, IR, and SSC regions. Nucleotide variation was higher in the LSC and SSC than the IR regions, as IR regions have low nucleotide diversity. There were three highly nucleotide-divergent regions, called mutational hotspots, located in the LSC (A) and SSC (B, C) regions, showing a Pi value of >0.03 (Figure 5; Supplementary Materials, Table S6). The first hotspot, A, covered the whole *trn*C-GCA gene; the second hotspot, B, was located on the *ycf*1 gene; while the third hotspot, C, consisted of the *rpl*32 gene and the intergenic spacer region *ndh*F–*rpl*32.

### 3.5. Phylogenetic Analysis

**Figure 6:**
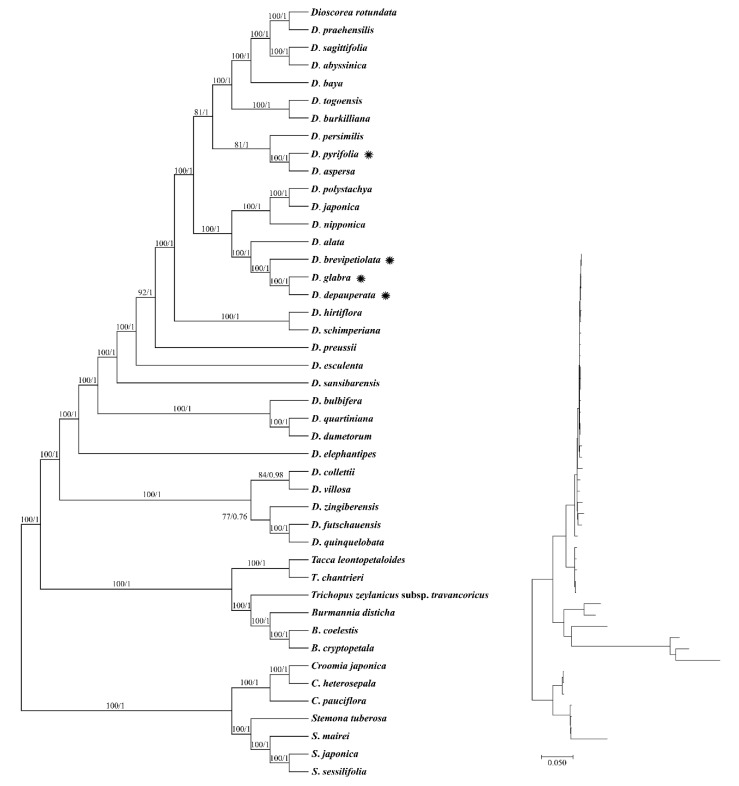
Phylogenetic trees inferred from maximum likelihood and Bayesian inference, showing genetic relationships of cp genome sequences of 37 species representing four different genera (*Burmannia*, *Dioscorea*, *Tacca*, and *Trichopus*) of Dioscoreales. Seven taxa of Pandanales, representing two genera (*Croomia* and *Stemona*), were included as an outgroup. The numbers associated with each node are bootstrap support values for ML (**left**) and posterior probability values for BI (**right**). Asterisks denote studied species.

As both the ML and BI trees displayed similar topology, only the ML tree is shown (Figure 6). Based on the phylogenetic analysis reconstructed using the complete cp genome sequences, a completely resolved phylogenetic relationship was recorded among species of *Dioscorea* for the ML tree, but not for the BI tree. Divergence is considered reliable when the bootstrap support (BS) value is equal to or more than 75%, while the posterior probability (PP) value is equal to or more than 0.90, as indicated on the branch node. By placing the seven Pandanales taxa as an outgroup, in Dioscoreales, the *Dioscorea* clade was sister to the *Burmannia* + *Tacca* + *Trichopus* clade. In the *Dioscorea* clade, two distinct groups can be observed—one of the groups contains five species, including *D. collettii*, *D. futchauensis*, *D. quinquelobata*, *D. villosa*, and *D. zingiberensis*, while all the other species were placed in the other group. A moderate PP value (PP = 0.76) was observed on the branch of the BI tree between the *D*. *futschauensis* + *D*. *quinquelobata* clade and *D*. *zingiberensis*. However, this branch was supported by the ML tree, in which a BS value of 77% was recorded. Based on current circumscription, *Dioscorea* exhibited a monophyletic relationship. A distinct divergence was recorded at the root of the *Dioscorea* clade, of which five species, including *D*. *collettii*, *D*. *futschauensis*, *D*. *quinquelobate*, *D*. *villosa*, and *D*. *zingiberensis*, formed a group that was separated from the other members of *Dioscorea*. For the four species of *Dioscorea* used in this study, *D*. *depauperata* was closely related to *D*. *glabra*, and they were clustered with two other species, where *D*. *alata* was first to diverge, followed by *D*. *brevipetiolata*. *D*. *pyrifolia* was closely related to *D*. *aspersa*, and both of them formed a group with *D*. *persimilis*.

## 4. Discussion

In this study, the cp genomes of four *Dioscorea* species that are native to Thailand were sequenced and assembled, and a comprehensive comparative analysis of these cp genomes was performed using other published cp genomes of the same genus obtained from NCBI GenBank. The cp genome sizes and characteristics of the four studied *Dioscorea* species, *D*. *brevipetiolata*, *D*. *depauperata*, *D*. *glabra*, and *D*. *pyrifolia*, are within a range that is similar to other reported cp genomes of *Dioscorea*, for which the complete cp genome sequence length is between 152,039 bp (*D. burkilliana*; GenBank no. MG805605) and 155,406 bp (*D. rotundata*; GenBank no. KJ490011). Within Dioscoreaceae, members of *Tacca* (GenBank nos. KX171420 and KT719235) have a larger cp genome size when compared to *Dioscorea*, which is approximately 163,000 bp, while the cp genome size of *Trichopus zeylanicus* subsp. *travancoricus* (GenBank no. MK674169) was 153,497, which is similar to that of *Dioscorea*. The repeat sequences found in the cp genome are products of the rearrangement and recombination of sequences in the cp genome [53]. Long repeat sequences play a role in inducing indels and identifying mutational hotspots [54], while SSRs are potentially useful in the characterization of closely-related species, as well as genetic differentiation at an intraspecific level, due to their high variability and reproducibility [55]. Based on our findings, we were unable to identify any patterns that could correlate the cp genome size and structure with the number of repeat sequences found. On the other hand, the finding from the IR border analysis somehow suggested that chloroplast genome evolution in *Dioscorea* seems to be highly conserved; the sequence length of the IR regions was similar, between 25,213 bp (*D. schimperiana*; GenBank no. MG805614) and 25,591 bp (*D. collettii*; GenBank no. KY996495). The expansion and contraction of the IR region allowed the movement of several genes adjacent to the junctions, including the *rps*19 and *ndh*F genes, to cross into the neighboring region. Although expansion and contraction of the IR region are common in the plant cp genome, they can differ in some degree [56]. Yet, the movement of genes crossing over the border in *Dioscorea* seems to not be drastic, suggesting that the evolution of the IR region in *Dioscorea* could be in its beginning stage.

Based on the finding from mVISTA, similar results of divergent regions have been previously reported in *Dioscorea* cp genomes, including *ndh*F, *ycf*1, *trn*K-*trn*Q, *trn*S-*trn*G, *trn*C-*pet*N, *trn*E-*trn*T, *pet*G-*trn*W-*trn*P, and *trn*L-*rpl*32 [22]. Moreover, the divergent regions include *trn*K-*trn*Q, *trn*S-*trn*G, *trn*C-*pet*N, *trn*E-*trn*T, *pet*G-*trn*W-*trn*P, and *trn*L-*rpl*32, where previous reports found that these divergent regions were mostly present in the SSC and LSC regions and showed a trend toward more rapid evolution [22,57,58,59]. With that in mind, DNA markers in the form of indels and nucleotide repeats could also be explored for species discrimination of *Dioscorea*. For example, two indel markers were developed from the complete cp genomes of six *Ipomoea* species [60], and five species-specific indel markers were developed to authenticate five species of *Panax* [61]. With at least eight different variable regions found in the alignment of the 25 cp genome sequences, based on mVISTA, as well as hundreds of repeats identified in the cp genome of *Dioscorea*, with several species of *Dioscorea* as important resources in traditional medicine production [62], novel indel and repeat markers could be developed to aid in species identification and authentication of these important species.

In a previous work, Zhao et al. [22] identified eight highly variable regions from a sliding window analysis of the cp genome sequences of nine species of *Dioscorea*. Among these eight highly variable regions, the *ycf*1 gene was also reported in our work, but the regions *trn*C, *rpl*32, and *ndh*F-*rpl*32, reported in our study, are new information. The difference in the discovery of novel hotspot regions may be due to the number of cp genome sequences used during the analysis; Zhao et al. [22] utilized nine species of *Dioscorea*, while 25 species of *Dioscorea* are included in this study. Altogether there is no study that evaluates the minimum cp genome sequences that should be included in a sliding window analysis to ensure high accuracy in hotspot detection, taxon sampling from eight to ten is recommended in search of a specific barcode [63]. Yet, an increase in taxon sampling may improve the accuracy of sequence alignment, which will further affect the information of variable sites delivered [64]. Therefore, we do not exclude the possibility that the hotspot regions identified in our study might be superior to those proposed by Zhao et al. [22] in terms of phylogenetic resolution at the species level. However, further experiments to verify the discrimination strength of these regions are required.

To our knowledge, this is the first work on phylogenetic tree reconstruction of *Dioscorea* that involved 31 different species, based on the complete cp genome sequence. Evidently, the use of the complete cp genome sequence in phylogenetic tree reconstruction of complicated genera has been recommended by many researchers, as it could yield promising results [65,66]. For example, the molecular placement of *D*. *aspersa*, *D*. *glabra*, and *D*. *persimilis* was ambiguous when using five cp and two mitochondrial DNA sequences [67], but was resolved in this study. In the same study, the phylogenetic tree, reconstructed using 48 *Dioscorea* taxa, revealed similar topology when compared to the phylogenetic tree based on the complete cp genome sequences. The divergence of the five species in our study complimented the grouping of the taxa from the section *Stenophora* [67]. The section *Stenophora* is recognized as the most basal clade in the phylogeny of *Dioscorea* [68], while the genus was proposed with more than 23 sections, with differing opinions being put forward. Nonetheless, a fully resolved phylogenetic tree was obtained in this study; it is recommended that an acceptable sample size ought to be achieved prior to phylogenetic reconstruction for taxonomic classification purposes. Although there is literature proposing the use of the complete cp genome sequence as super-barcodes that are effective in delimiting closely related species [69], performing NGS on a large number of samples might not be favorable to some laboratories due to sequencing cost and availability of sequencing facilities. Thus, identifying a powerful DNA region that is adequate for phylogenetic analysis of *Dioscorea*, as suggested in the previous paragraph on the DNA barcoding of *Dioscorea*, is deemed requisite.

## 5. Conclusions

The genomic data generated in this study can be potentially useful for the authentication of *Dioscorea* species, and can be further developed into powerful species-specific markers of *Dioscorea* species, using both subtle details and the overall cp genome. Additionally, beyond reducing the necessary research time, funding, and the number of plant species studied, the findings from the phylogenetic analysis of *Dioscorea* based on the complete cp genome sequences have provided much insight into the molecular placement and phylogenetic relationship among the members of *Dioscorea* used in this study. Further taxonomic classification of *Dioscorea* should also consider the use of this NGS dataset for reconstruction of phylogenetic trees at the genome level, to aid in combing out the taxonomic uncertainties among these complicated species.

## Figures and Tables

**Table 1:** General characteristics of complete chloroplast genomes of the four *Dioscorea* species.

Sample Name	Total Length (bp)	GC (%)	LSC Region Length (bp)	SSC Region Length (bp)	IR Region Length (bp)	Protein–Coding Genes	Transfer RNA Genes	Ribosomal RNA Genes	GenBank Accession Number
*D*. *brevipetiolata*	153,485	37	83,720	18,813	25,476	79	30	4	OL638495
*D*. *depauperata*	153,487	37	83,710	18,825	25,476	79	30	4	OL638496
*D*. *glabra*	153,503	37	83,724	18,827	25,476	79	30	4	OL638497
*D*. *pyrifolia*	153,370	37	83,692	18,886	25,396	79	30	4	OL638498

**Table 2:** List of genes, including their function, groups, and names, in the four *Dioscorea* species chloroplast genomes.

Function of Gene	Group of Gene	Gene Name
Photosynthesis related genes	Assembly and stability of Photosystem I	* *ycf*3, *ycf*4
	ATP synthase	*atp*A, *atp*B, *atp*E, * *atp*F, *atp*H, *atp*I
	cytochrome b/f compelx	*pet*A, * *pet*B, * *pet*D, *pet*G, *pet*L, *pet*N
	cytochrome c synthesis	*ccs*A
	NADPH dehydrogenase	* *ndh*A, * *ndh*B (2), *ndh*C, *ndh*D, *ndh*E, *ndh*F, *ndh*G, *ndh*H, *ndh*I, *ndh*J, *ndh*K
	Photosystem I	*psa*A, *psa*B, *psa*C, *psa*I, *psa*J
	Photosystem II	*psb*A, *psb*B, *psb*C, *psb*D, *psb*E, *psb*F, *psb*H, *psb*I, *psb*J, *psb*K, *psb*L, *psb*M, *psb*N, *psb*T, *psb*Z
	Rubisco	*rbc*L
Transcription and translation related genes	ribosomal proteins	*rps*2, *rps*4, *rps*3, *rps*7 (2), *rps*8, *rps*11, * *rps*12 (2), *rps*14, *rps*15, *rps*18, *rps*19, * *rpl*2 (2), *rpl*14, * *rpl*16, *rpl*20, *rpl*22, *rpl*23 (2), *rpl*32, *rpl*33, *rpl*36
	ribosomal RNA	*rrn*4.5 (2), *rrn*5 (2), *rrn*16 (2), *rrn*23 (2)
	transcription	*rpo*A, *rpo*B, * *rpo*C1, *rpo*C2
	transfer RNA	* *trn*A-UGC (2), *trn*C-GCA, *trn*D-GUC, *trn*E-UUC, *trn*F-GAA, *trnf*M-CAU, *trn*G-GCC, *trn*H-GUG (2), *trn*I-CAU (2), * *trn*I-GAU (2), * *trn*K-UUU, *trn*L-CAA (2), * *trn*L-UAA, *trn*L-UAG, *trn*M-CAU, *trn*N-GUU (2), *trn*P-UGG, *trn*Q-UUG, *trn*R-ACG (2), *trn*R-UCU, *trn*S-GCU, *trn*S-GGA, *trn*S-UGA, * *trn*T-CGU, *trn*T-GGU, *trn*T-UGU, *trn*V-GAC (2), * *trn*V-UAC, *trn*W-CCA, *trn*Y-GAU
	translation initiation factor	*inf*A
Other genes	carbon metabolism	*cem*A
	fatty acid synthesis	*acc*D
	proteolysis	* *clp*P
	RNA processing	*mat*K
Genes of unknown function	conserved reading frames	*ycf*1, *ycf*2 (2), *ycf*15 (2)

* = Gene with intron; (2) = 2 repeat units.

## Data Availability

The complete chloroplast genome sequences of the four *Dioscorea* species were submitted at NCBI (GenBank accession number: OL638495–OL638498).

## Supplementary Materials

The following supporting information can be downloaded at: https://www.mdpi.com/article/10.3390/genes14030703/s1, Table S1: The GenBank accession numbers and the names of samples using in chloroplast genome analysis; Table S2: Gene information of four *Dioscorea* species choloplast genomes; Table S3: Genes with introns in chloroplast genomes of four *Dioscorea* species; Table S4: Repeat analysis of the four *Dioscorea* species chloroplast genomes; Table S5: Simple sequence repeats in four *Dioscorea* species chloroplast genomes; Table S6: The nucleotide diversity values of 25 *Dioscorea* species.

## Abbreviations

Chloroplast genome	cp
Large single copy	LSC
Inverted repeats	IRs
Small single copy	SSC
Coding sequence	CDS
