# Supplementary material for: Complete Chloroplast Genome of Four Thai Native Dioscorea Species: Structural, Comparative and Phylogenetic Analyses
Source: Genes (Basel). 2023 Mar 12;14(3):703. doi: 10.3390/genes14030703 (PMC10048501; doi:10.3390/genes14030703)
Supplement: Supplementary file 1 [file genes-14-00703-s001.zip › Supplementary information.pdf]

**Supplementary information**

Table S1: The GenBank accession numbers and the names of samples using in chloroplast genome analysis; Table S2: Gene information of four *Dioscorea* species choloplast genomes; Table S3: Genes with introns in chloroplast genomes of four *Dioscorea* species; Table S4: Repeat analysis of the four *Dioscorea* species chloroplast genomes; Table S5: Simple sequence repeats in four *Dioscorea* species chloroplast genomes; Table S6: The nucleotide diversity values of 25 *Dioscorea* species.
